# The Diagnostic Value of CEUS in Assessing Non-Ossified Thyroid Cartilage Invasion in Patients with Laryngeal Squamous Cell Carcinoma

**DOI:** 10.3390/jcm13030891

**Published:** 2024-02-03

**Authors:** Milda Pucėtaitė, Davide Farina, Silvija Ryškienė, Dalia Mitraitė, Rytis Tarasevičius, Saulius Lukoševičius, Evaldas Padervinskis, Saulius Vaitkus

**Affiliations:** 1 Department of Radiology, Medical Academy, Lithuanian University of Health Sciences, A. Mickevičiaus Str. 9, 44307 Kaunas, Lithuania; silvija.ryskiene@lsmu.lt (S.R.); dalia.mitraite@lsmu.lt (D.M.); saulius.lukosevicius@lsmu.lt (S.L.); 2 Department of Radiological Sciences, University of Brescia, Piazzale Spedali Civili 1, 25123 Brescia, Italy; davide.farina@unibs.it; 3 Department of Radiology, Lithuanian University of Health Sciences Kaunas Clinics, Eivenių 2, 50009 Kaunas, Lithuania; rytis.tarasevicius@kaunoklinikos.lt; 4 Department of Otorhinolaryngology, Medical Academy, Lithuanian University of Health Sciences, A. Mickevičiaus Str. 9, 44307 Kaunas, Lithuania; evaldas.padervinskis@lsmu.lt (E.P.); saulius.vaitkus@lsmu.lt (S.V.)

**Keywords:** non-ossified thyroid cartilage, CEUS, CECT, MRI, laryngeal cancer

## Abstract

**Background:** Accurate assessment of thyroid cartilage invasion in squamous cell carcinoma (SCC) of the larynx remains a challenge in clinical practice. The aim of this study was to assess the diagnostic performance of contrast-enhanced ultrasound (CEUS), contrast-enhanced computed tomography (CECT), and magnetic resonance imaging (MRI) in the detection of non-ossified thyroid cartilage invasion in patients with SCC. **Methods:** CEUS, CECT, and MRI scans of 27 male patients with histologically proven SCC were evaluated and compared. A total of 31 cases were assessed via CEUS and CECT. The MR images of five patients and six cases were excluded (one patient had two suspected sites), leaving twenty-five cases for analysis via MRI. **Results:** CEUS showed the highest accuracy and specificity compared with CECT and MRI (87.1% vs. 64.5% and 76.0% as well as 84.0% vs. 64.0% and 72.7%, respectively). The sensitivity and negative predictive value of CEUS and MRI were the same (100%). CEUS yielded four false-positive findings. However, there were no statistically significant differences among the imaging modalities (*p* > 0.05). **Conclusions:** CEUS showed better diagnostic performance than CECT and MRI. Therefore, CEUS has the potential to accurately assess non-ossified thyroid cartilage invasion and guide appropriate treatment decisions, hopefully leading to improved patient outcomes.

## 1. Introduction

Imaging of the local spread of laryngeal cancer plays an important role in choosing a suitable treatment strategy, such as organ-sparing therapy, radical surgery, or combined therapy. The decision regarding which treatment strategy to employ affects the effectiveness of treatment and quality of life [1,2,3]. The role of imaging is more crucial in discriminating between T3 and T4 stages than between T1 and T2 stages according to the tumor-node-metastasis (TNM) classification. As reported in the review by Deganello et al. [4], patients with T4 stage tumors have a higher risk of developing lymph node metastases, which also affects both prognosis and treatment planning. Therefore, radiologists often face great challenges in evaluating subtle findings.

Both contrast-enhanced computed tomography (CECT) and magnetic resonance imaging (MRI) are the main and most widely used modalities for laryngeal imaging; most guidelines leave the choice between the two techniques up to local protocols and scanner availability. In CECT, one of the most controversial issues in the assessment of the tumor invasion of non-ossified thyroid cartilage is a similar post-contrast density of the tumor and the non-ossified thyroid cartilage [5]. In these cases, dual-energy computed tomography (DECT) or MRI may provide added value. In particular, the study published by Becker et al. [6] demonstrated that the application of revised MRI criteria led to an overall statistically significant improvement in the assessment of thyroid cartilage invasion. However, none of the cross-sectional techniques outperform the others on the specific issue of non-ossified thyroid cartilage [5,6,7,8,9,10].

Contrast-enhanced ultrasound (CEUS) can be used to assess and quantify microcirculation in normal and pathological conditions with a good acoustic window [11]. Moreover, it has been widely used in clinical practice to diagnose hepatic and renal pathologies [12,13]. In a recent publication, CEUS showed potential in assessing non-ossified thyroid cartilage invasion [14]. Non-ossified thyroid cartilage and adjacent laryngeal cancer are well visualized on CEUS due to the differences between non-enhancing non-ossified thyroid cartilage and enhancing adjacent laryngeal cancer. Therefore, the detection of enhancement along the course of a thyroid lamina contacting a tumor suggests infiltration. However, there is currently a lack of studies and data that can strongly support the use of CEUS as an additional imaging modality in the diagnostic algorithms for determining the local spread of laryngeal cancer more accurately. 

Therefore, the purpose of this study was to assess the diagnostic value of CEUS, compared with that of CECT and MRI, in the detection of non-ossified thyroid cartilage invasion in SCC of the larynx.

## 2. Materials and Methods

### 2.1. Patients 

Between 2021 and 2023, a prospective comparative study was carried out at the Hospital of Lithuanian University of Health Sciences Kaunas Clinics. A total of 38 patients with histopathologically proven SCC of the larynx were enrolled in this study. The inclusion criteria were as follows: an available CECT scan demonstrating pathological infiltration adjacent to the non-ossified tract of the thyroid cartilage or its clear infiltration; no history of previous laryngeal–hypopharyngeal surgery or chemoradiation; and having undergone surgery planned after multidisciplinary team discussion. Eleven patients were excluded because they refused surgical treatment or did not attend further consultations or undergo further surgery.

All 27 male patients meeting the inclusion criteria were subjected to CEUS and MRI. Informed consent was obtained from all participants before the study. The study was conducted according to the guidelines of the Declaration of Helsinki. Ethical approval was obtained from Kaunas Regional Biomedical Research Ethics Committee (protocol No. 2021-BE-10-00016; dated 2021).

### 2.2. CECT Examination

Multislice CT examinations were performed using an Aquilion ONE TSX-301 scanner (Toshiba, Tokyo, Japan) with the following parameters: 120 kVp; specific effective mAs for each patient based on the patient’s size and tissue thickness; collimation, 128 × 0.625 mm; field of view, 260 mm; and matrix, 512 × 512. The patients were asked to assume a supine position, breathe quietly, and avoid coughing and swallowing. The field of view was from the skull base to the aortic arch. Scanning was performed without and with intravenous contrast media (65–100 mL) with a 50 mL saline flush to obtain contrast-enhanced images with a 60–80 s delay after administration; the concentration of iodine in the contrast agent was 320–370 mg/mL. Images were reconstructed for axial (parallel to the plane of the true vocal cords), sagittal, and coronal (perpendicular to the plane of the true vocal cords) planes with soft tissue and bone algorithms (2 mm in thickness). 

### 2.3. CEUS Examination

CEUS examination was performed using a Philips Epiq 7 (expert-class) US system (Philips Healthcare, Best, The Netherlands) with a 5–12-MHz linear transducer. The patients were asked to assume the supine position with their necks extended. The larynx and its surrounding structures were evaluated in the transverse and longitudinal sections. The distance between the area of lesion contact to the non-ossified thyroid cartilage seen via CECT and the upper border of the thyroid lamina was measured via CECT and then used as a reference to target the same area through CEUS.

CEUS examination was performed by administering an intravenous bolus of SonoVue (Bracco SpA, Milan, Italy) (5 mL, followed by saline flush) [14]. The scan was performed with a frequency of 12 MHz and a mechanical index of 0.08. The patients were asked to refrain from swallowing and coughing during the examination. Dynamic perfusion of the tumor and peritumoral tissues was observed and recorded in the hard drive of the device for about 1 min. If there was more than one suspected site of invasion, the CEUS procedure was repeated after 10 min. 

### 2.4. MRI Examination

MRI examination was performed using a Philips Ingenia 3.0T scanner (Philips Healthcare, Best, The Netherlands) with dedicated head–neck 20-channel parallel imaging array coils. The patients were imaged in the supine position and asked to breathe quietly and refrain from swallowing and coughing during the scanning. Axial images were captured parallel to the plane of the true vocal cords; coronal images were obtained perpendicular to this plane. The MRI protocol employed is specified in Table 1. 

### 2.5. Image Analysis

The analysis of CEUS images was performed by two radiologists with >4 and >20 years of experience, respectively. The findings from the CECT and MRI examinations were interpreted by one head-and-neck radiologist with >20 years of experience in head-and-neck imaging. The radiologists were not blinded to the clinical and CECT information during the analysis of CEUS and MRI images.

#### 2.5.1. CEUS Imaging

**Figure 1:**
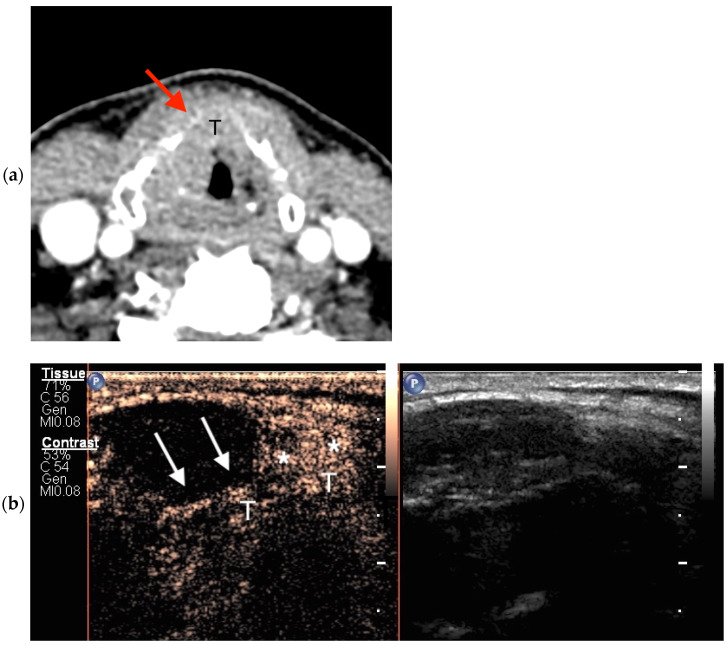
True-positive findings through axial CECT and CEUS. (**a**) In this CECT image, a partial bilateral ossification of the thyroid cartilage with a similar tissue density between the tumor (T) and the non-ossified thyroid cartilage (red arrow) can be seen; (**b**) CEUS image taken after intravenous contrast material administration showing the enhancement of the tumor (T) with invasion of the right anterior part of the non-ossified thyroid cartilage (asterisks); the adjacent hypoechogenic cartilage is non-invaded (white arrows).

CEUS images were evaluated and interpreted by both radiologists during examination and post-processing. The non-ossified thyroid cartilage was considered infiltrated by a tumor when contrast enhancement was observed (Figure 1). When the cases were evaluated, there was no disagreement between the radiologists.

#### 2.5.2. Cross-Sectional Imaging

CECT and MRI images were evaluated and interpreted according to previous articles [6,7,9,15].

**Figure 2:**
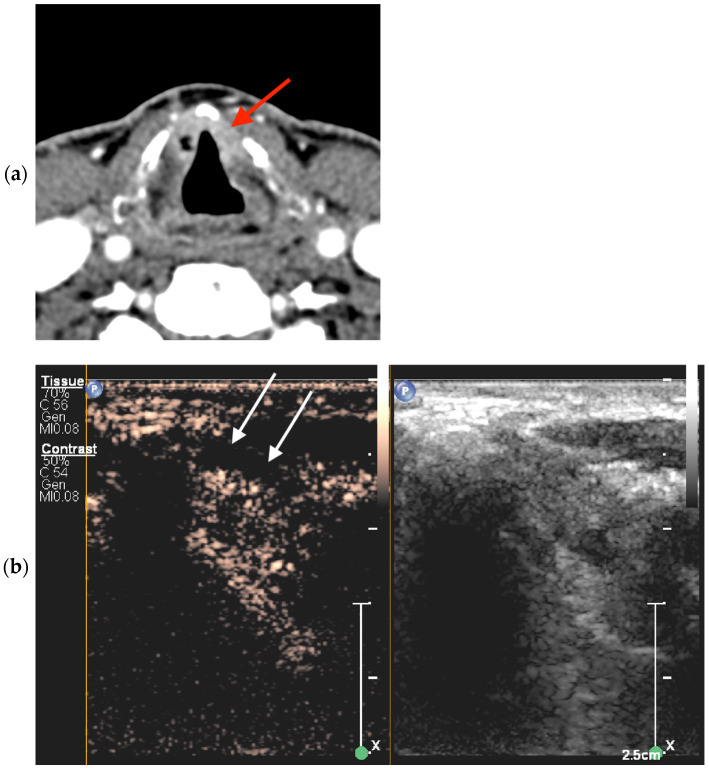
Bilateral glottic cancer adjacent to the non-ossified thyroid cartilage lamina. (**a**) Axial CECT findings on the left side were false-positive for tumor invasion (red arrow). (**b**) CEUS image of the left side at the same level as (**a**) in the transverse plane shows true-negative findings, i.e., non-enhanced non-ossified cartilage (white arrows).

In CECT images, non-ossified thyroid cartilage invasion was positive when the following criteria were met: a focal cartilage defect in close proximity to the tumor was found; replacement of the cartilage by soft tissue with enhancement matching that adjacent to the cartilage occurred; and the lesion was in direct contact with the thyroid cartilage and densities were indistinguishable (Figure 2). Findings obtained via CECT were considered negative if the densities between the tumor/pathologic infiltration and the non-ossified cartilage were distinguishable. 

**Figure 3:**
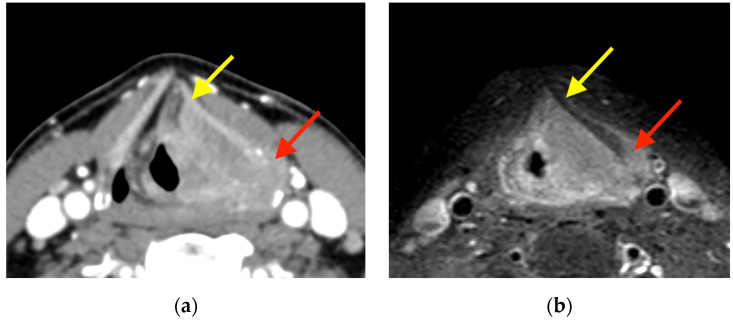

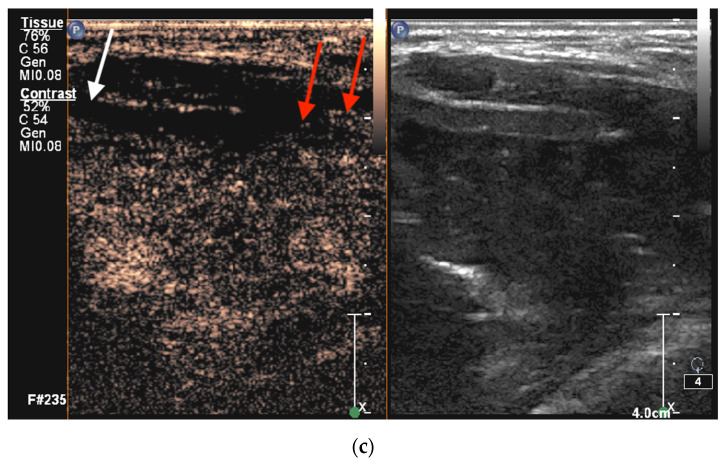
Supraglottic squamous cell carcinoma on the left side. (**a**) Axial CECT represents two sites, namely, sites that were false-positive anteriorly (yellow arrow) and true-positive posteriorly (red arrow), whereas MRI (**b**) contrast-enhanced high-resolution T1-weighted turbo spin echo Dixon and CEUS (**c**) findings were true-negative anteriorly (white arrow) and true-positive posteriorly (red arrows), respectively.

When conducting MRI, thyroid cartilage invasion was diagnosed when the thyroid lamina showed abnormal signal intensity matching the signal of the tumor in T2-weighted image (T2WI), T1-weighted image (T1WI) (before and after contrast administration), DWI, and ADC map (Figure 3). When the thyroid lamina showed a T2WI signal, enhancement, and an ADC value higher than those of the tumor, the abnormal signal was classified as inflammation.

### 2.6. Histologic Examination

A pathologist with >20 years of experience evaluated the surgical specimens according to the existing guidelines described elsewhere [16]. To ensure precise correspondence between radiological findings and pathology, the suspected area of invasion was indicated by radiologists on an anatomical sketch of the larynx that accompanied each specimen.

### 2.7. Statistical Analysis

The IBM SPSS Statistics 20.0 (IBM Corp. in Armonk, NY, USA) statistical software package was used in this study. Sensitivity, specificity, accuracy, negative predictive value (NPV), and positive predictive value (PPV) of CEUS, CECT, and MRI in evaluating non-ossified laryngeal cartilage involvement were assessed by comparing results with histopathological findings [17,18]. Accuracy was calculated according to the following formula:

$$Accuracy=\frac{TP+TN}{TP+TN+FP+FN}$$

where TP is true positive; TN denotes true negative; FP denotes false positive; and FN denotes false negative. 

McNemar’s test was used to compare the accuracy of imaging modalities. A *p* value of <0.05 was considered statistically significant.

## 3. Results

In this prospective study, 27 male patients with a mean age of 63 years (SD, 8.7; range, 46–84 years) were enrolled.

Overall, there were 31 cases, as four patients had two suspected sites of non-ossified thyroid cartilage invasion. All 31 cases were assessed using CEUS and CECT. The MR images of five patients (corresponding to 6 cases, as one patient had two suspected sites) were non-diagnostic due to major artifacts, leaving 25 cases for analysis via MRI. 

There were 14 cases (51.9%) of glottic SCC and 13 cases (48.1%) of transglottic SCC with the majority showing a G2 degree of differentiation (85.2%). The patients’ distribution by pT staging is shown in Table 2. 

**Figure 4:**
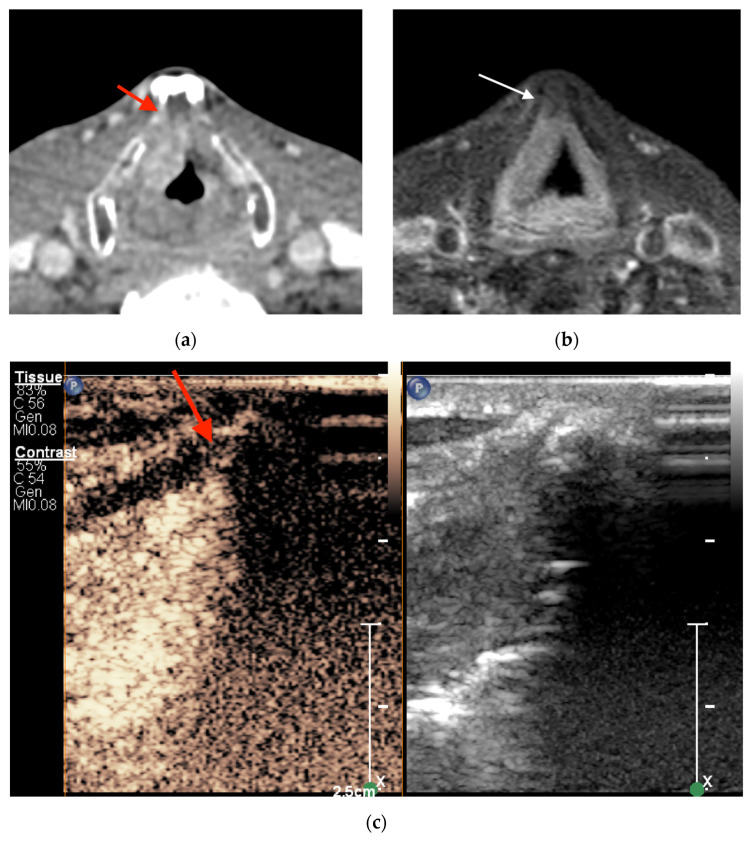
Supraglottic squamous cell carcinoma on the right side anteriorly adjacent to non-ossified cartilage inner lamina. (**a**) Axial CECT and (**c**) CEUS findings were false positive (red arrow) for tumor invasion of the thyroid cartilage, whereas MRI findings, as shown in (**b**), in axial contrast-enhanced high-resolution T1-weighted turbo spin echo Dixon images were true negative (white arrow).

In six cases (19.4%), histological proof of non-ossified thyroid cartilage invasion was obtained. The diagnostic performance of imaging studies is shown in Table 3. There were no statistically significant differences among the modalities (*p* > 0.05). CEUS and MRI showed a NPV of 100%. CEUS had four false-positive findings (Figure 4); however, the PPV was higher than those of CECT and MRI (60% vs. 30.8% and 33.3%, respectively).

There were no statistically significant differences between these imaging modalities (*p* > 0.05). CEUS, contrast-enhanced ultrasound; CECT, contrast-enhanced computed tomography; MRI, magnetic resonance imaging; TP, true positive; TN, true negative; FP, false positive; FN, false negative; PPV, positive predictive value; NPV, negative predictive value.

## 4. Discussion

In the current study, we aimed at evaluating the diagnostic performance of CEUS, CECT, and MRI in detecting non-ossified thyroid cartilage tumor invasion, taking postoperative histopathological examination as the gold standard. Our results show that based on the presence of enhancement, CEUS allows for the discrimination of invaded (i.e., enhancing) from normal (i.e., non-enhancing) non-ossified thyroid cartilage. CEUS, CECT, and MRI evaluation demonstrated high accuracy (87.1%, 64.5%, and 76%, respectively) with minor differences. Moreover, CEUS was slightly superior to other modalities employed in this study in detecting non-ossified thyroid cartilage tumor invasion. 

The detection of laryngeal cartilage invasion can significantly influence the choice of optimal treatment strategy and the prognosis of SCC of the larynx. Currently, the choice of optimal treatment strategy is controversial. However, in the case of thyroid cartilage invasion or its suspicion, transoral laryngeal microsurgery (TOLMS) should be ruled out due to possible non-radical tumor removal, and in such cases, open partial horizontal laryngectomy (OPHL), total laryngectomy, or non-surgical treatments should be considered [2,3,19,20,21]. In addition, deep tumor invasion into the thyroid cartilage leads to negative outcomes through treatment with radiation therapy [21]. Therefore, for the selection of an optimal treatment plan avoiding complications and incomplete resection as well as improving disease control and survival, an accurate clinical and radiological assessment of local spread, especially the most controversial invasion of the cartilage, is necessary. 

Cross-sectional imaging with multi-slice CT or MRI is designed to map deep tumor spread to the submucosal soft tissues and cartilaginous framework. CECT examination can be quickly performed, is widely available, and allows volumetric acquisition with a submillimetric voxel size: the short acquisition time minimizes the risk of motion artifacts, while the high spatial resolution allows the detection of subtle areas of tumor invasion of soft tissue spaces and cartilage [9,22,23].

MRI has higher contrast resolution, which is boosted by the possibility of combining different pulse sequences. In the literature, this potential has mainly been exploited to assess cartilage invasion [6,24], and MRI is reported to have significantly higher sensitivity than CECT for cartilage invasion [22]. A recent meta-analysis of studies involving patients with laryngo–hypopharyngeal cancer reported pooled sensitivities of 88% for MRI and 66% for CT, with specificities of 81% and 90%, respectively, in the detection of cartilage invasion [22]. Expectedly, CT’s performance was more heterogeneous than that of MRI, as it differed when taking into account which type of cartilage was involved: when only thyroid cartilage was analyzed, the sensitivity was 69%, which was close to that in our study (66%), but the specificity was higher (86% vs. 64%). Based on the results of the above-mentioned meta-analysis and our current study, we can assume that the diagnosis of thyroid cartilage invasion poses significant challenges that are better handled by MRI than CT. 

However, most studies evaluating the diagnostic performance of imaging techniques in the detection of cartilage invasion tend to focus—intentionally or unintentionally—on the ossified cartilage. This occurs for several reasons: first, because, in most cases, invasion involves the ossified parts, and second, because CT and MRI better visualize the invasion of ossified cartilage, manifesting with a panel of findings including sclerosis, erosion, or destruction with cartilage replacement by tumor tissue [5]. This is mainly due to the lack of differences in density in CT images between the tumor and the non-ossified thyroid cartilage and because of the overlapping features of the tumor and non-neoplastic changes such as reactive inflammation, edema, and fibrosis in MRI images [22,24]. Peritumoral inflammation is another potential confounding factor at the interface between a tumor and cartilage, although the combination of different sequences may improve differentiation when conducting MRI.

DECT is another promising imaging modality that has been analyzed in recent years. One research group [25] used DECT to evaluate the spectral attenuation curves of tumor tissue and non-ossified thyroid cartilage. Virtual monochromatic images (VMIs) of different energy levels showed that tumor tissue density dropped in higher-kiloelectron-volt VMIs, while the non-ossified cartilage maintained high attenuation, allowing distinguishment between the two. However, this study did not directly evaluate non-ossified cartilage invasion by tumor tissue. 

US was also previously investigated for its possible role in solving the problem of thyroid cartilage invasion. Indeed, US seemed to uniquely take a place among cross-sectional modalities for evaluating non-ossified cartilage invasion, as the larynx is a superficial structure, and because it best visualizes the non-ossified parts, which present the most diagnostic challenges when conducting CT and MRI scans [14,26,27]. One study involving 62 patients with laryngeal or hypopharyngeal cancer showed that US and CECT had sensitivities of 98% and 91%, respectively, and equal specificities of 75% [26]. The authors speculated that clear visualization of the fat plane between the tumor and the cartilage as well as independent movement of the thyroid cartilage and adjacent tumor tissues contributed to the higher sensitivity of US [26]. The results of the previously mentioned studies prompted the cited researchers to further analyze the possibilities of US examination by incorporating CEUS.

Our study aligns with the study by Hu et al. [14] in terms of showing a higher accuracy of CEUS than CECT (90% and 83%, respectively) in detecting thyroid cartilage invasion, even though there were the following relevant differences in the methodological part: In our study, the exact site of possible non-ossified thyroid cartilage invasion as indicated by CECT examination was further investigated using CEUS and MRI and postoperative histopathological examination. Moreover, the radiologist who carried out the CEUS and MRI examinations and the pathologist were not blinded to the CECT findings. To the best of our knowledge, this is the first study comparing CEUS, CECT, and MRI regarding the specific topic of non-ossified cartilage invasion. Although we did not observe statistically significant differences among the three imaging modalities (*p* > 0.05), based on the promising results, we suggest that CEUS may be considered a usable imaging modality complementary to CECT and MRI for the assessment of non-ossified thyroid cartilage in the non-infrequent event of equivocal CECT and/or MRI findings. One of the limitations of this study is its small sample size. A second limitation is that the CECT and MRI images were assessed by a single expert/observer. Moreover, in our practice, in some cases, matching the suspected site seen in CECT images to the site observed in CEUS images was difficult due to the small region of interest. In the future, this issue could be solved by fusing CECT with US, and this should be performed by a head-and-neck radiologist due to their comprehensive knowledge of laryngeal anatomy and CECT imaging. Moreover, only one region of interest can be investigated at a time; therefore, we reinjected a contrast agent for the evaluation of another site of tumor invasion, leading to the extended examination time. 

## 5. Conclusions

CEUS showed slightly higher diagnostic values in the detection of non-ossified thyroid cartilage invasion in laryngeal and hypopharyngeal cancer than CECT and MRI. This may result in CEUS being an important problem-solving tool in routine clinical practice that can be used to confidently assess non-ossified thyroid cartilage invasion and guide appropriate treatment decisions, hopefully leading to improved patient outcomes. Further studies are needed to increase the number of observations and confirm the evidence obtained.

## Figures and Tables

**Table 1:** MRI protocol.

Sequence	Plane	Slice Thickness, mm	Repetition Time, ms	Time to Echo, ms	Field of View, mm
High-resolution T2-weighted turbo spin echo Dixon	Axial, coronal, sagittal	2.5–3	2888	80	190–210
High-resolution T1-weighted turbo spin echo Dixon	Axial	2.1–2.5	634	8	190–210
DWI and ADC	Axial	2	14,439; 220	66	250
Contrast-enhanced high-resolution T1-weighted turbo spin echo Dixon	Axial, coronal	2.1–2.5	634	8	190–210

DWI, diffusion-weighted imaging; ADC, apparent diffusion coefficient.

**Table 2:** Distribution of the patients according to pT staging.

pT Group	n (%)
pTis	1 (3.7)
pT1	7 (25.9)
pT2	7 (25.9)
pT3	8 (29.6)
pT4	4 (14.8)

Staging was performed according to the American Joint Committee on Cancer/Union for International Cancer Control (AJCC/UICC), 8th Edition, guidelines.

**Table 3:** Diagnostic performance of CEUS, CECT, and MRI in the assessment of non-ossified thyroid cartilage invasion.

Imaging Modality	TP, n	TN, n	FP, n	FN, n	Sensitivity, % (95% CI)	Specificity, % (95% CI)	Accuracy, % (95% CI)	PPV, %	NPV, %
CEUS (n = 31)	6	21	4	0	100.0 (54.1–100.0)	84.0 (63.2–95.5)	87.1 (70.2–96.4)	60.0	100.0
CECT (n = 31)	4	16	9	2	66.7 (22.3–95.7)	64.0 (42.5–82.0)	64.5 (45.4–80.8)	30.8	88.9
MRI (n = 25)	3	16	6	0	100.0 (29.2–100.0)	72.7 (49.8–89.3)	76.0 (54.9–90.6)	33.3	100.0

## Data Availability

The data that support the findings of this study are available from the corresponding author upon reasonable request. The data are not publicly available due to privacy or ethical restrictions.
